# The NAILED stroke risk factor trial (Nurse based Age independent Intervention to Limit Evolution of Disease after stroke): study protocol for a randomized controlled trial

**DOI:** 10.1186/1745-6215-14-5

**Published:** 2013-01-05

**Authors:** Thomas Mooe, Lisa Bergström, Anna-Lotta Irewall, Joachim Ögren

**Affiliations:** 1 Department of Public Health and Clinical Medicine, Umeå University, Östersund, Sweden; 2 Department of Internal Medicine, Section of Neurology, Östersund Hospital, Östersund, Sweden; 3 Department of Internal Medicine, Section of Cerebrovascular Diseases, Östersund Hospital, Östersund, Sweden; 4 Department of Internal Medicine, Section of Cardiology, Östersund Hospital, Östersund, Sweden

**Keywords:** Stroke, Secondary prevention, Cardiovascular diseases, Randomized controlled trial

## Abstract

**Background:**

Secondary prevention after stroke and transient ischemic attack (TIA) is essential in order to reduce morbidity and mortality. Secondary stroke prevention studies have, however, been fairly small, or performed as clinical trials with non-representative patient selection. Long-term follow-up data is also limited. A nurse-led follow-up for risk factor improvement may be effective but the evidence is limited. The aims of this study are to perform an adequately sized, nurse-led, long-term secondary preventive follow-up with a population-based inclusion of stroke and TIA patients. The focus will be on blood pressure and lipid control as well as tobacco use and physical activity.

**Methods:**

A randomized, controlled, long-term, population-based trial with two parallel groups. The patients will be included during the initial hospital stay. Important outcome variables are sitting systolic and diastolic blood pressure, LDL cholesterol and total cholesterol. Outcomes will be measured after 12, 24 and 36 months of follow-up. Trained nurses will manage the intervention group with a focus on reaching set treatment goals as soon as possible. The control group will receive usual care. At least 200 patients will be included in each group, in order to reliably detect a difference in mean systolic blood pressure of 5 mmHg. This sample size is also adequate for detection of clinically meaningful group differences in the other outcomes.

**Discussion:**

This study will test the hypothesis that a nurse-led, long-term follow-up after stroke with a focus on reaching set treatment goals as soon as possible, is an effective secondary preventive method. If proven effective, this method could be implemented in general practice at a low cost.

**Trial registration:**

Current Controlled Trials ISRCTN23868518

## Background

The risk of stroke recurrence, myocardial infarction and death after stroke is high [1,2]. Secondary prevention after stroke is essential in order to reduce morbidity and mortality, consequently leading to reduced costs to society. There are a number of evidence based treatment strategies for secondary prevention after stroke [3]. Risk factor modification may reduce the risk of stroke recurrence by up to 80% [4]. Despite these facts, secondary stroke prevention studies have mostly been performed as clinical trials with non-representative patient selection, for example concerning age and co-morbidity [5]. There is considerable literature about non-pharmacological interventions but a paucity of data concerning the implementation of proven pharmacological interventions in patients representative of clinical praxis [6]. The number of long-term follow-up studies of secondary prevention after stroke is, to date, limited.

The implementation of risk factor intervention after stroke is difficult. There are limitations in terms of compliance and the proportion of patients achieving set treatment goals in usual care [5,7]. Even though adherence to prescribed medication may be quite high in some cohorts of stroke patients [8], the proportion achieving set treatment goals among the various risk factors has been less studied. A strategy to improve secondary prevention results by early interventions shows potential [9]. On the other hand, there is evidence that even high quality secondary preventive measures initiated while patients are hospitalized are no guarantee of a successful outcome after discharge [10]. Further research in this area is essential.

There is limited evidence that nurse-led follow up for risk factor improvement after stroke has a positive outcome [11]. In order to improve secondary prevention after stroke, we have designed a trial with the following components:

1. Population-based. All patients in the county of Jämtland, Sweden, admitted to the county hospital with transient ischemic attack (TIA) or stroke will be considered for inclusion in the study, regardless of age, gender or co-morbidity.

2. The study will be randomized with a nurse-led intervention group and a control group being seen by their general practitioner for usual medical care.

3. The intervention is based on telephone follow-ups by a stroke nurse, in order to facilitate implementation and minimize the necessary resources.

4. A routine of promptly adjusting medication in order to achieve set treatment goals for secondary stroke prevention will be established.

5. Duration of the study. A follow-up with risk factor evaluation after 12, 24 and 36 months is planned.

In this nurse-based telephone follow-up study, we hypothesized that the outcome, in terms of risk factor control, would be better in the intervention group.

Ethics approval has been received from the Ethics Committee, Umea University.

This paper presents the design of the study according to the CONSORT requirements [12].

## Methods/design

### Trial design

A randomized, controlled trial with two parallel groups and an allocation ratio of 1:1.

### Participants

All patients living in the county of Jämtland, Sweden, who were hospitalized with a diagnosis of stroke (ischemic or hemorrhagic) or TIA will be assessed for inclusion. Östersund hospital is the only hospital in the county and all patients, those in terminal care excluded, with symptoms of a suspected TIA or stroke, are referred here for diagnostic evaluation. It is a rural catchment area with a population of approximately 125,000 inhabitants. A routine for identification of all patients in the hospital with a possible TIA or stroke, based on performed brain computed tomography examinations, has been established. During a three-month test period the study nurses identified all patients with a final TIA or stroke diagnosis. All patients physically and mentally capable of communicating by telephone will be eligible. This means that patients suffering from aphasia, dementia and deafness will be excluded. The other exclusion criteria are severe, often terminal, disease, and participation in another on-going trial.

### Interventions

All eligible patients will be informed about the study and asked to give a written informed consent. They will receive standard information about stroke concerning the pathophysiology and risk factors according to established clinical praxis during their hospitalization. They will also be offered a follow-up visit to a stroke nurse and an outpatient follow-up according to normal care procedures.

Patients randomized to the intervention group will be contacted by a study nurse by phone one month after discharge. Before the call, a blood sample for lipids will be taken and a standardized blood pressure control will be performed. Blood pressure will be measured after five minutes in the sitting position and after one minute standing. The tests will be performed by a district nurse, or, for patients in the intervention group living close to the hospital, by a study nurse. Self-reported compliance with medication will be recorded. During the call the patient will be informed about the test results and whether a change in medication is necessary. Tobacco use, physical activity and dietary habits will be discussed. Smoking cessation will be encouraged and supported. Physical activity of moderate intensity for 30 minutes or more, most days of the week will be encouraged, but also adjusted to the individual patient’s capacity. Dietary advice to reduce saturated fat and increase the intake of fruit and vegetables will be given. If the patient’s cholesterol or blood pressure values are higher than anticipated, medication will be adjusted after contact from a study physician. Repeated tests will be taken within approximately four weeks and further adjustments made if necessary until target values are reached or no further changes can be considered realistic. The same routine, with an Hb1C test added, will be applied after 12, 24 and 36 months.

The target values are: blood pressure <140/<90 mmHg (optionally <130/<80 mmHg in patients at very high risk, that is, diabetic subjects), total cholesterol <4.5 mmol/l, LDL <2.5 mmol/l [13,14].

**Figure 1:**
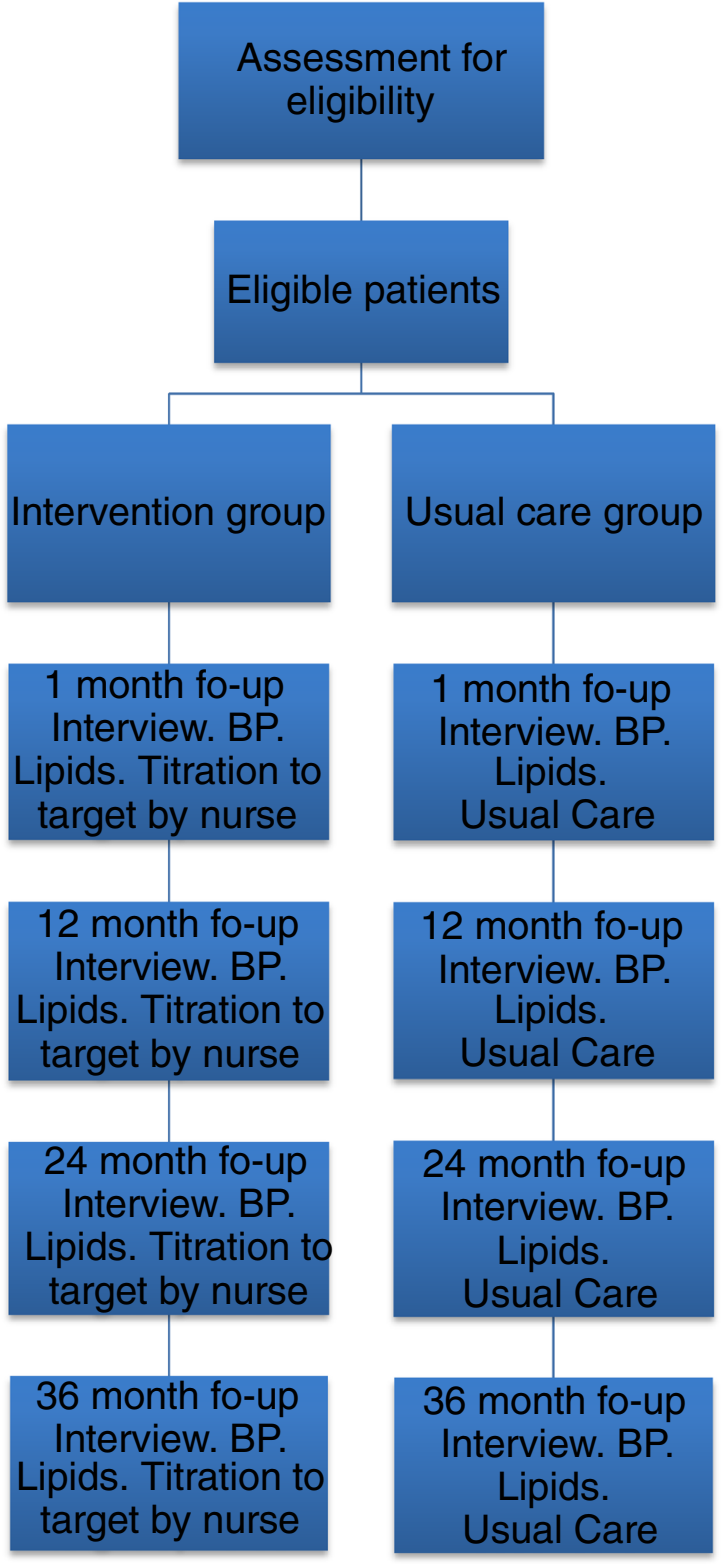
**Study flow chart.** BP = blood pressure measurement.

Patients randomized to the usual care group will also be contacted by phone one month following discharge after blood pressure and lipid profile have been checked. Self-reported compliance, tobacco use and physical activity will be recorded. All medical care will be given by their treating physician, usually a general practitioner, who will receive the test results (lipid profile and blood pressure), and no additional intervention will be given as a result of participation in the study. The same routine, with an Hb1C test added, will be applied after 12, 24 and 36 months, Figure 1.

### Outcomes

Outcomes will be measured after 12, 24 and 36 months of follow-up. Outcome variables are sitting systolic and diastolic blood pressure, LDL cholesterol and total cholesterol, as well as the proportion of patients achieving the set target for these variables. Standing systolic and diastolic blood pressure, smoking rates, the proportion of patients treated with different secondary preventive drugs, diabetes control by Hb1C, change in body mass index (BMI) and physical activity will also be measured. Sitting systolic blood pressure at 36 months will be analyzed as the primary outcome. The analyses of the secondary outcomes will be exploratory.

Blood pressures measurements are standardized as described above. LDL values are calculated from the serum concentrations of cholesterol and fasting triglycerides using the Friedewald formula. Smoking (yes/no) and physical activity (duration/week) are self-reported. Deaths are available in the hospital records and will be recorded in order to detect any survival difference between groups.

### Sample size

To reliably detect a difference between groups in mean systolic blood pressure of 5 mmHg requires study groups of 180 participants (standard deviation 19, mean systolic blood pressure 140 versus 135, alpha 0.05 two-tailed, power 80%). Study groups of at least 200 participants are planned to allow for drop-outs. This sample size is also adequate for detection of clinically meaningful group differences in lipid values (0.3 mmol/l, standard deviation 1.0), smoking rates (10%), proportion reaching treatment goals (10%), change in BMI (1.0, standard deviation 4.0) and change in physical activity (10%, proportion in a given activity level), calculated with two-tailed alpha 0.05, power 80%.

### Randomization

The random allocation sequence will be computer generated in blocks of four and stratified for gender and degree of disability (modified Rankin scale <3 and ≥3). A sealed, colored envelope will have a serial number on the outside and a folded sheet of paper inside with the group allocation written on it: this will be impossible to read from the outside. The study coordinators will enroll participants and assign them to interventions in order according to the serial number. The random allocation sequence will be computer generated by the study manager who is not involved in the randomization process. Each month, the correct use of envelopes will be controlled for ten patients.

### Statistical methods

The primary analysis will be performed according to the intention-to-treat principle using a linear regression model adjusting for gender and degree of disability. The adjustment is made in order to reflect the stratified randomization process. Per protocol analyses will also be performed. Secondary outcomes will be analyzed using the primary analysis model when continuous, and using a logistic regression model, adjusting for the same covariates, when outcomes are binary. In order to assess whether there are indications of differential treatment effects across subgroups (age, gender, co-morbidity, level of education and social classification), tests for interaction will be performed, although this aim is secondary because the study is not powered for this particular purpose. Adjustment for relevant baseline covariates will be performed in additional exploratory analyses of primary and secondary outcomes in order to evaluate the effect of possible baseline imbalance. All tests will be two-sided and a *P*-value of <0.05 will be considered significant.

## Discussion

Stroke is one of the major diseases in the current ageing population, with substantial consequences for individuals and society. The knowledge of secondary preventive measures to prevent further disease in stroke patients is strikingly low. Further research in the areas of compliance, the possibility of participation in secondary preventive studies and the possibility of reaching treatment goals in a cohort of stroke patients representative for clinical praxis is absolutely necessary.

This study covers all patients in one Swedish county, who after TIA or stroke, will be eligible for inclusion, thus avoiding selection bias. This allows us to study even very old and very sick stroke patients, who are usually not included in randomized stroke studies. Their ability to participate in secondary prevention programs and their tolerability of medication when attempting to achieve set treatment goals are of great interest as these have significant consequences for health economics and mortality and morbidity rates.

The study design has several other advantages. Randomization of the patients soon after the stroke or TIA allows us to study the acute phase as well as to perform a long-term follow-up. By establishing an early contact, no patient is expected to be lost to follow-up. The immediate recruitment and the logistics to include each and every stroke and TIA patient will show the true proportion of possible participants in the secondary preventive program.

As it is well known that information and team rehabilitation after stroke affects the outcome, the study is designed so that each eligible patient will receive the same present standard of care regardless of randomization group.

Our intention is to combine a nurse-led life-style intervention with a rapid pharmaceutical titration model aiming to achieve set treatment goals of the most important risk factors after stroke. The long-term follow-up and the relatively large number of patients involved will provide important new data in the field of improving secondary prevention after stroke. Cost-effectiveness of this method of handling stroke follow-up can be studied, and we are of the opinion that this method - if proven effective - could be implemented in general clinical practice at a low cost. The study logistics are deliberately made as simple as possible in order to be generalizable to the Swedish as well as to other national health care systems. Although the study is performed in a single county, the information on better control of standard risk factors is also likely to be valid in other health care systems.

## Trial status

Ongoing.

## Abbreviations

TIA: Transient ischemic attack; BMI: Body mass index; LDL: Low-density lipoprotein.

## Competing interests

The authors have no competing interests.

## Authors’ contributions

TM conceived and designed the research. TM and LB drafted the initial manuscript. LB, ALI and JÖ were involved in the critical revision for important intellectual content and assisted with writing the final manuscript. All authors read and approved the final manuscript.

## References

[B1] Mohan KM Wolfe CD Rudd AG Heuschmann PU Kolominsky-Rabas PL Grieve AP Risk and cumulative risk of stroke recurrence: a systematic review and meta-analysis Stroke 2011 42 5 1489 1494 10.1161/STROKEAHA.110.602615 21454819

[B2] Touze E Varenne O Chatellier G Peyrard S Rothwell PM Mas JL Risk of myocardial infarction and vascular death after transient ischemic attack and ischemic stroke: a systematic review and meta-analysis Stroke 2005 36 12 2748 2755 10.1161/01.STR.0000190118.02275.33 16254218

[B3] Rothwell PM Algra A Amarenco P Medical treatment in acute and long-term secondary prevention after transient ischemic attack and ischemic stroke Lancet 2011 377 9778 1681 1692 10.1016/S0140-6736(11)60516-3 21571151

[B4] Hackam DG Spence JD Combining multiple approaches for the secondary prevention of vascular events after stroke: a quantitative modeling study Stroke 2007 38 6 1881 1885 10.1161/STROKEAHA.106.475525 17431209

[B5] Furie KL Kasner SE Adams RJ Albers GW Bush RL Fagan SC Halperin JL Johnston SC Katzan I Kernan WN Guidelines for the prevention of stroke in patients with stroke or transient ischemic attack: a guideline for healthcare professionals from the American heart association/American stroke association Stroke 2011 42 1 227 276 10.1161/STR.0b013e3181f7d043 20966421

[B6] MacKay-Lyons M Thornton M Ruggles T Manley S Non-pharmacological interventions for preventing secondary vascular events after stroke or transient ischemic attack (Protocol) Cochrane Database Syst Rev 2010 Issue 9 CD008656 10.1002/14651858.CD008656 PMC11813163 23543566

[B7] Alvarez-Sabin J Quintana M Hernandez-Presa MA Alvarez C Chaves J Ribo M Therapeutic interventions and success in risk factor control for secondary prevention of stroke J Stroke Cerebrovasc Dis 2009 18 6 460 465 10.1016/j.jstrokecerebrovasdis.2009.01.014 19900649

[B8] Bushnell CD Olson DM Zhao X Pan W Zimmer LO Goldstein LB Alberts MJ Fagan SC Fonarow GC Johnston SC Secondary preventive medication persistence and adherence one year after stroke Neurology 2011 77 12 1182 1190 10.1212/WNL.0b013e31822f0423 21900638 PMC3265047

[B9] Ovbiagele B Saver JL Fredieu A Suzuki S Selco S Rajajee V McNair N Razinia T Kidwell CS In-hospital initiation of secondary stroke prevention therapies yields high rates of adherence at follow-up Stroke 2004 35 12 2879 2883 10.1161/01.STR.0000147967.49567.d6 15514170

[B10] Ross JS Arling G Ofner S Roumie CL Keyhani S Williams LS Ordin DL Bravata DM Correlation of inpatient and outpatient measures of stroke care quality within veterans health administration hospitals Stroke 2011 42 8 2269 2275 10.1161/STROKEAHA.110.611913 21719771 PMC3144276

[B11] Adie K James MA Does telephone follow-up improve blood pressure after minor stroke or TIA? Age Ageing 2010 39 5 598 603 10.1093/ageing/afq085 20667838

[B12] Schulz KF Altman DG Moher D Group C CONSORT 2010 Statement: updated guidelines for reporting parallel group randomized trials BMC Med 2010 8 1 18 10.1186/1741-7015-8-18 21686296 PMC3116666

[B13] Mancia G Laurent S Agabiti-Rosei E Ambrosioni E Burnier M Caulfield MJ Cifkova R Clement D Coca A Dominiczak A Reappraisal of European guidelines on hypertension management: a European Society of Hypertension Task Force document J Hypertens 2009 27 11 2121 2158 10.1097/HJH.0b013e328333146d 19838131

[B14] Graham I Atar D Borch-Johnsen K Boysen G Burell G Cifkova R Dallongeville J De Backer G Ebrahim S Gjelsvik B European guidelines on cardiovascular disease prevention in clinical practice: executive summary. Fourth Joint Task Force of the European Society of Cardiology and other societies on cardiovascular disease prevention in clinical practice (constituted by representatives of nine societies and by invited experts) Eur J Cardiovasc Prev Rehabil 2007 14 Suppl 2 E1 E40 17726406 10.1097/01.hjr.0000277984.31558.c4

